# Deep cervical lymphaticovenous anastomosis for Alzheimer's disease: A narrative review

**DOI:** 10.1002/alz.71038

**Published:** 2025-12-29

**Authors:** Qingwen Chen, Qianmei Wen, Tao Zhong, Jian Liu, Han Gao

**Affiliations:** ^1^ Department of Neurosurgery The Affiliated Qingyuan Hospital (Qingyuan People's Hospital) Guangzhou Medical University Qingyuan China; ^2^ Department of Neurosurgery the First Affiliated Hospital of Guangdong Pharmaceutical University Guangzhou China; ^3^ State Key Laboratory of Respiratory Diseases National Clinical Research Center for Respiratory Diseases Guangzhou Institute of Respiratory Health the First Affiliated Hospital of Guangzhou Medical University Guangzhou China

**Keywords:** Alzheimer's disease, amyloid beta, deep cervical lymphaticovenous anastomosis, glymphatic system, surgical treatment, tau

## Abstract

Alzheimer's disease (AD) is a common neurodegenerative disorder with limited treatment options. Recent discoveries of the glymphatic system and meningeal lymphatic vessels (MLVs) have highlighted their critical role in clearing metabolic waste—including amyloid beta and tau proteins—from the brain. Dysfunction of these systems contributes to AD pathogenesis by impairing the clearance of neurotoxic proteins. Deep cervical lymphaticovenous anastomosis (dcLVA) is an innovative microsurgical technique designed to enhance cerebral waste drainage by anastomosing deep cervical lymphatic channels to adjacent veins. This narrative review synthesizes current evidence on the mechanisms, applications, and emerging perspectives of dcLVA for AD. Early studies suggest potential short‐term improvements in cognitive scores and neuroimaging biomarkers after surgery, with an acceptable safety profile. However, the evidence is limited to small prospective cohorts and case reports, underscoring the need for larger, randomized controlled trials to validate its efficacy and long‐term benefits. dcLVA represents a promising surgical intervention for select patients, particularly those with moderate‐to‐severe AD who have failed conventional pharmacotherapy, but it requires careful patient selection and further investigation.

**Highlights:**

- This review proposes deep cervical lymphaticovenous anastomosis (dcLVA) as a mechanism driven surgery that may enhance clearance of amyloid beta (Aβ) and tau through the brain, meningeal, and deep cervical drainage pathways.
- Early human evidence from a single arm cohort and case reports suggests short term cognitive and imaging signals with acceptable perioperative safety.
- dcLVA is not recommended as a first line option for early Alzheimer's disease (AD) and may be considered for moderate to severe AD or for patients who are refractory to pharmacotherapy and have objective evidence of drainage impairment.
- Standardized patient selection and longitudinal imaging and biomarker monitoring are recommended, including Aβ and tau positron emission tomography (PET), diffusion tensor imaging (DTI) analysis along the perivascular space, and cerebrospinal fluid (CSF) or plasma panels.
- Systemic safety remains an important uncertainty, and future trials should include longitudinal surveillance of hepatic, renal, and hematologic function.
- Multicenter randomized controlled trials (RCTs) are urgently needed with 12 month Clinical Dementia Rating Sum of Boxes (CDR SB) as a primary endpoint, transparent reporting, and evaluation of combination strategies with anti Aβ therapies.

## 1 INTRODUCTION

Alzheimer's disease (AD) is the most common neurodegenerative disease and the leading cause of dementia, accounting for ≈ 60% to 80% of all dementia cases.^1^ Recent epidemiological data indicate that roughly 57.4 million people worldwide have dementia, the vast majority of whom have AD, and this number is projected to reach 152.8 million by 2050.^2^ In China, a country with a large population, there are currently ≈ 17 million AD patients (≈ 30% of the global prevalence), and this figure is rapidly increasing with population aging.^3^ Available treatment strategies are extremely limited, relying primarily on four symptom‐managing drugs: three acetylcholinesterase inhibitors (donepezil, galantamine, and rivastigmine) and one N‐methyl‐D‐aspartic acid (NMDA) receptor antagonist (memantine).^4^ These medications can only temporarily relieve symptoms, have limited therapeutic effects, and cannot halt disease progression.^4^, ^5^, ^6^, ^7^ Therefore, exploring new therapeutic strategies for AD is an urgent challenge.

**FIGURE 1:**
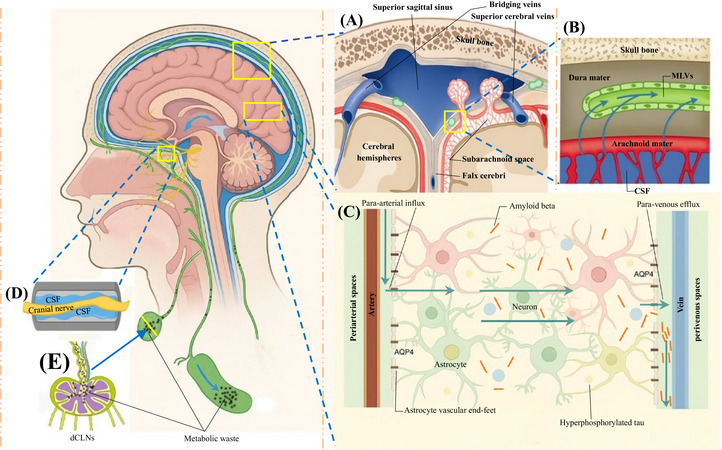
Schematic diagram of MLVs, the glymphatic system, and metabolic waste clearance. A, B, MLV drainage. The glymphatic system drains metabolic waste from the brain parenchyma to the MLVs, and CSF carrying waste is actively taken up by MLVs and transported to dCLNs. C, Structural diagram of the glymphatic system. After CSF enters the brain parenchyma along the PVS around arteries, it is transported to the brain tissue via AQP4 on astrocyte endfeet, exchanges substances with the brain ISF, and the mixed fluid is transported via AQP4 to the perivenous spaces. D, CSF passes through bony channels along the perineural spaces of cranial nerves, enters the submucosal lymphatic vessels of the nose, and finally drains to dCLNs. E, The drained metabolic waste enters dCLNs. AQP4, aquaporin‐4; CSF, cerebrospinal fluid; dCLN, deep cervical lymph node; ISF, interstitial fluid; MLV, meningeal lymphatic vessel.

The discovery of the brain's lymphatic‐like system has opened new avenues for treatment. In 2012, Iliff et al. first systematically described the glymphatic system, a core network for clearing metabolic waste from brain parenchyma, composed mainly of perivascular spaces (PVSs), aquaporin‐4 (AQP4) water channels on astrocyte endfeet, and perivenous spaces.^8^ This system allows cerebrospinal fluid (CSF) to enter brain tissue along periarterial spaces, mix with interstitial fluid (ISF), and clear waste via paravenous routes (Figure 1C).^8^ In 2015, Aspelund et al. and Louveau et al. independently confirmed the existence of dural meningeal lymphatic vessels (MLVs; Figure 1A, B), lymphatic channels in the dura mater that express lymphatic endothelial markers and drain CSF and ISF to deep cervical lymph nodes (dCLNs; Figure 1E).^9^, ^10^ In addition to dural meningeal lymphatic drainage, CSF can also exit the cranial cavity along perineural pathways accompanying cranial nerves and subsequently enter extracranial lymphatics, providing an additional route to the cervical lymphatic system (Figure 1D).^81^ Subsequently, Absinta et al. visualized similar structures in living humans and non‐human primates using high‐resolution magnetic resonance imaging (MRI), further validating the presence of a central lymphatic system in humans.^11^ These discoveries overturned the traditional view that the central nervous system (CNS) lacks lymphatic drainage and provided a new theoretical framework for studying the pathology of AD and other neurodegenerative diseases.^12^

The glymphatic system and MLVs together form a complete “brain–lymphatic clearance axis” responsible for removing metabolic waste from the brain, including toxic proteins such as amyloid beta (Aβ) and tau.^13^ Studies have shown that dysfunction of this clearance axis is a key link in AD pathology: loss of AQP4 polarization leads to a 40% to 60% reduction in CSF–ISF exchange efficiency, while degeneration of MLVs manifests as slowed drainage and narrowed vessel caliber.^14^, ^15^, ^16^ These changes together greatly reduce waste clearance efficiency, promoting formation of amyloid plaques and neurofibrillary tangles. Animal experiments have confirmed that disruption of MLVs or deletion of AQP4 can reduce Aβ clearance by 65%, significantly exacerbate Aβ/tau accumulation, and provoke microglial activation, astrocyte proliferation, and cognitive decline.^15^ Conversely, interventions that promote lymphangiogenesis—such as intrathecal injection of vascular endothelial growth factor‐C (VEGF‐C) to expand MLVs or the use of prostaglandin F2α to enhance cervical lymphatic contractility—have been shown to reduce pathological protein burden and ameliorate AD pathology.^17^, ^18^ Clinical studies suggest that glymphatic dysfunction in AD may precede abnormal Aβ deposition and is closely associated with neurodegeneration and cognitive decline. For example, the diffusion tensor image analysis along the perivascular space (DTI‐ALPS) index, an imaging marker of glymphatic function, is already reduced in patients with mild cognitive impairment (MCI) or prodromal dementia.^19^ Sleep is also a critical regulator of the glymphatic system: during non–rapid eye movement slow‐wave sleep, glymphatic flow is markedly enhanced, and sleep disturbances are closely linked to an increased risk of AD and dementia.^20^, ^21^ Chronic sleep deprivation (≤ 6 hours per night) in people > 50 years old increases dementia risk by ≈ 30%.^22^ These findings indicate that targeting pathways of waste clearance based on the mechanisms of glymphatic dysfunction may provide new therapeutic strategies for AD.

Based on the critical role of the glymphatic–meningeal lymphatic system in AD pathology, an innovative minimally invasive surgical therapy, deep cervical lymphaticovenous anastomosis (dcLVA), has been gradually applied in clinical practice for AD patients.^23^ In this procedure, deep cervical lymphatic channels are anastomosed to adjacent veins to restore and improve brain lymphatic drainage pathways, thereby reducing the accumulation of pathological proteins like Aβ and tau in the brain. In this review, we comprehensively analyze and synthesize the existing literature to evaluate the theoretical basis, technical features, clinical efficacy, and future directions of this surgical approach.

## 2 CLASSIFICATION OF LVA PROCEDURES

### 2.1 dcLVA

dcLVA is an innovative procedure based on supermicrosurgical techniques. Its theoretical foundation arises from the development of classical LVA techniques and the growing understanding of the brain's lymphatic clearance system. Traditional LVA techniques developed in the latter half of the twentieth century, primarily for treating lymphedema.^24^, ^25^, ^26^ With advances in neuroscience demonstrating the crucial role of the brain lymphatic system in clearing CNS metabolic waste, Professor Xie Qingping's team pioneered the application of LVA to AD treatment and proposed dcLVA specifically targeting the deep cervical lymphatic system.^23^ In dcLVA, deep cervical lymphatic vessels (typically <0.8 mm in diameter) are precisely anastomosed to neighboring veins, reconstructing and enhancing lymphatic drainage pathways from the brain. This procedure aims to reduce accumulation of pathological proteins in the brain and provides a new theoretical foundation and technical approach for the surgical treatment of neurodegenerative diseases.^23^, ^27^, ^28^ Detailed technical aspects and features of dcLVA are discussed below.

### 2.2 Other LVA procedures

Several other LVA‐related procedures exist, but due to the complexity of cervical anatomy, these have not been applied to AD treatment. These include lymph node–rich tissue transplantation (LFT), vascularized lymph node transfer (LNT), and lymphatic biomaterial–venous anastomosis. These techniques have greater technical difficulty, more complex protocols, and higher potential complication rates. Compared to dcLVA, their cost‐to‐benefit ratio may be less favorable. Table 1 summarizes the status and features of these other LVA procedures. Notably, these approaches are currently used mainly for limb lymphedema and have not been attempted for enhancing intracranial lymphatic drainage.

**TABLE 1:** LVA procedures not yet applied in the treatment of patients with AD.

Procedure	Surgical features	Clinical applications	Current status
**LFT**	Transfer a pedicled or free flap rich in lymphatic tissue (lymphatic vessels/microscopic lymph nodes) to the recipient site. A typical approach is to harvest an adipocutaneous flap containing multiple lymphatic vessels (e.g., an abdominal superficial circumflex iliac artery perforator flap) together with its supplying artery and vein, transplant it en bloc, and, when feasible, anastomose its lymphatic vessels to those of the recipient site.^69^ Unlike simple lymph node transplantation, LFT transplants tissue that contains an intact lymphatic network; meticulous preservation of donor‐site lymphatic outflow is required intraoperatively to avoid iatrogenic lymphedema.^48^	Primarily used for refractory lymphedema in which conventional lymphatic decompression procedures (e.g., LVA, LNT) are ineffective or not feasible. For example, Mihara et al. reported that a modified abdominal LFT for chronic lower‐limb lymphedema resulted in a marked reduction in limb circumference within 1 year (maximum decrease of 13.5 cm).^69^	No reports or studies targeting AD; this technique is applied to reconstruct peripheral (limb) lymphatic drainage, and there have been no attempts to use it to improve cerebral lymphatic outflow.
**Vascularized LNT**	Under microsurgical technique, a vascularized, intact lymph node flap is transplanted to the edematous region. Common donor sites include the groin, supraclavicular fossa, and occipital region. Intraoperatively, the lymph node and its nourishing artery and vein are transferred en bloc to the recipient site; end‐to‐end anastomoses are performed for the artery and vein, and the nodal pedicle vessels are anastomosed to the recipient vessels.^70^, ^71^ After transplantation, the lymph node can promote lymphangiogenesis via surrounding capillary lymphatics, helping to reconstruct lymphatic outflow. The procedure is complex and time consuming, requiring microsurgical anastomosis of small vessels.	Indicated for limb lymphedema at any stage—especially moderate‐to‐severe or fibrotic disease.^71^ Commonly reported indications include postmastectomy upper‐limb lymphedema and postpelvic cancer lower‐limb lymphedema. Literature reviews show that LNT can significantly reduce edema severity and symptoms (average limb‐volume reduction ≈ 30%–50%), with particular benefit in markedly fibrotic cases.^70^, ^71^ In recent years, combined intraoperative LNT + LVA and preventive ILR have also been explored.	No applications have been reported for AD. LNT is used primarily to reconstruct peripheral lymphatic drainage; studies applying vascularized lymph node transfer to improve cerebral lymphatic outflow are not yet available.
**Lymphatic biomaterial–venous anastomosis**	Uses artificial materials to establish lymphatic bypasses or to promote lymphatic regeneration. Examples include subcutaneous implantation of silicone tubing to create a drainage conduit under the limb,^72^ or implantation of a biocompatible scaffold (e.g., the nanofibrillar collagen scaffold BioBridge) to guide neolymphangiogenesis and recanalization.^73^ A relatively new technique: Çevirme et al. reported placing antibiotic‐coated silicone tubing within a subcutaneous tunnel as an “artificial lymphatic channel” adjunct in patients with advanced lower‐limb lymphedema.^72^ Nguyen et al. applied a collagen scaffold in patients who had previously undergone LVA/VLNT and observed greater formation of new lymphatic collectors and enhanced lymphatic efflux.^73^	Used mainly for refractory stage III/IV lymphedema in which conventional minimally invasive procedures are ineffective. Silicone‐tube methods can augment compression therapy, markedly improving limb contour and reducing edema postoperatively.^72^ The BioBridge scaffold is often combined with LVA/VLNT, substantially increasing edema‐reduction rates and facilitating lymphatic return.^73^ Overall, biomaterial‐based strategies remain in the exploratory clinical phase with limited reports.	No related studies in AD to date. Biomaterials are used primarily to reconstruct peripheral lymphatic drainage; they do not involve surgical applications to the intracranial lymphatic system, and there have been no attempts or reports of their use for AD.

Abbreviations: AD, Alzheimer's disease; ILR, immediate lymphatic reconstruction; LFT, lymph node–rich tissue transplantation; LNT, lymph node transfer; LVA, lymphaticovenous anastomosis; VLNT, vascularized lymph node transfer.

## 3 CLINICAL APPLICATION OF dcLVA IN AD

Current evidence for using dcLVA to treat AD is still at an early exploratory stage, with few publications. Therefore, based on existing literature, we first outline the characteristics, advantages, and limitations of this technique, and compare it to other AD treatments. Below, we use tables and narrative discussion to describe the details of using dcLVA for AD treatment, including comparisons to other therapies and the status of clinical implementation. We hope that by summarizing current literature, future researchers will be guided in conducting related basic and clinical studies.

### 3.1 Indications, contraindications, and risk–benefit assessment of dcLVA

As described above, dcLVA creates a supermicrosurgical anastomosis between the drainage channels of the deep cervical lymph trunks/nodes and adjacent veins (such as branches of the internal/external jugular veins or small venules, typically 0.3–0.8 mm in diameter). This procedure reduces resistance to outflow at the extracranial terminus (deep cervical chain/venous angle), thereby enhancing the overall clearance efficiency of the “brain–meninges–deep cervical” pathway under the assumption that proximal pathways remain patent. In theory, this promotes the efflux of Aβ and tau and relieves neuroinflammation. The deep cervical lymph nodes serve as the final destination for CSF drainage, and their dysfunction can lead to accumulation of metabolic waste in the brain.^29^ However, it is important to emphasize that not all forms of “glymphatic dysfunction” are amenable to dcLVA. The surgery has potential benefit only when the surgically accessible bottleneck is primarily at the extracranial terminus and the meningeal/parenchymal lymphatic pathways are largely unobstructed. If extensive proximal clearance deficits exist (for example, significant blockage of meningeal lymphatic vessels or markedly insufficient glymphatic driving force), then creating a bypass in the neck alone may have no obvious effect.^13^, ^15^ Currently, however, there are no clear diagnostic criteria or screening methods to identify which AD patients have lymphatic drainage dysfunction amenable to dcLVA.

Based on available literature, we summarize the following potential indications for dcLVA, and Table 2 summarizes the core differences between dcLVA and other AD therapies. The preferred patient populations include:

1. Moderate‐to‐severe AD (Clinical Dementia Rating [CDR] ≥ 2): Priority is given to moderate‐to‐severe stages because, in early AD (MCI/mild dementia), there are already proven anti‐Aβ monoclonal antibodies (e.g., lecanemab, donanemab) whose indications are limited to “early AD.”^30^, ^31^ In such patients, standard guideline‐directed pharmacotherapy should be given priority, and invasive surgical interventions should not be first‐line. In contrast, for moderate‐to‐severe AD, clear evidence and indications for anti‐Aβ drugs are lacking; from an ethical and risk–benefit perspective, moderate‐to‐severe patients are more suitable for surgical feasibility and safety trials.
2. Patients with objective evidence of impaired brain lymphatic drainage after ≥ 6 months of standard therapy: For early AD patients who have received ≥ 6 months of standard therapy (including acetylcholinesterase inhibitors [donepezil, galantamine, and rivastigmine] and/or NMDA antagonist [memantine] and/or anti‐Aβ antibodies) and still experience deterioration beyond the minimal clinically important difference (MCID), dcLVA can be considered. For example, this could be defined as a Mini‐Mental State Examination (MMSE) decline of ≥ 1.4 to 3 points within 6 months (depending on disease stage) or a CDR Sum of Boxes (CDR‐SB) increase ≥ 2 points; functional scales like the Alzheimer's Disease Cooperative Study Activities of Daily Living (ADCS‐ADL) showing MCID changes can also confirm deterioration.^32^, ^33^, ^34^ Patients who cannot maintain therapeutic doses due to adverse reactions or contraindications (e.g., refractory bradycardia, persistent gastrointestinal side effects, and severe weight loss) and who have been fully evaluated by a multidisciplinary team may be considered to have failed medical therapy.^35^ In these patients, with confirmed impairment of brain–deep cervical lymphatic drainage, dcLVA may be an option. It is worth noting that confirming impaired brain–deep cervical drainage is based on our theoretical framework; currently, practical use of this criterion in surgical candidates or its description in the literature has not been observed.

**TABLE 2:** Clinical criteria for dcLVA in treating Alzheimer's disease and core differences from other therapies.

Key clinical criteria	dcLVA	Anti‐Aβ monoclonal antibody: Lecanemab	Anti‐Aβ monoclonal antibody: Donanemab	Cholinesterase inhibitors (donepezil/galantamine/rivastigmine)	NMDA receptor antagonist (memantine)
Applicable disease stage / scale thresholds	① Definite diagnosis of AD; ② Recommend MMSE ≤ 20 (indicating ≥ moderate impairment) as one surgical eligibility criterion; ③ Postoperative efficacy and follow‐up scales: MMSE, MoCA, CDR, ADAS‐Cog (note: use “improvement ≥ 4 points” on ADAS‐Cog as an effect criterion; not suitable for very mild or very severe disease)	Early AD: MCI due to AD or mild dementia; MMSE 22–30; typically 50–90 years	Early AD: MCI/mild dementia; MMSE 20–30	Usable in mild, moderate, and severe AD	Moderate‐to‐severe AD
Required biomarkers / diagnostic evidence	Diagnosis and staging per the latest NIA–AA clinical criteria: among the core biomarkers A (Aβ abnormality: decreased CSF Aβ42/40 ratio or amyloid PET positivity) and T (elevated p‐tau or tau PET positivity), abnormality in either core biomarker is sufficient to diagnose AD; disease severity is classified as mild–moderate–severe based on the degree of A/T abnormality	Evidence of Aβ positivity required (PET or CSF p‐tau/Aβ42 ratio, etc.)	Evidence of Aβ positivity required (PET or CSF); tau PET not mandatory	No mandatory Aβ or tau biomarker requirement (clinical diagnosis sufficient)	No mandatory Aβ or tau biomarker requirement (clinical diagnosis sufficient)
Imaging / testing prerequisites and monitoring	Preoperative: ① PET‐CT (FDG/FAPI/AV1451; AV1451 is tau PET) to assess Aβ/tau burden; ② MRI including hippocampal thin‐slice plus (if needed) DWI for atrophy and differential diagnosis; ③ ICG navigation to localize deep cervical and skull‐base lymphatic pathways; ④ Carotid CTA to exclude severe stenosis; ⑤ CSF/plasma Aβ42/40, p‐tau, t‐tau, NfL, GFAP; ⑥ *APOE* ε4 genotyping. Postoperative: screen for complications at 1 week; assess clinical improvement at 4 weeks; reassess scales at 3/6/12 months; at 3 months, PET CT or CSF retesting is recommended.	MRI within the 12 months prior to treatment; ARIA monitoring: recommended MRI before the 5th, 7th, and 14th infusions, with some patients re‐imaged again at 52 weeks; MRI protocol should include FLAIR, T2*GRE/SWI, DWI, etc.	MRI within the 12 months prior to treatment; ARIA monitoring: MRI before the 2nd, 3rd, 4th, and 7th infusions; for high‐risk patients, again before the 12th; additional scans as needed.	No specific imaging prerequisites; routine clinical follow‐up assessments are sufficient.	No specific imaging prerequisites; attend to renal function and potential drug–drug interactions during routine evaluation.
Major exclusions / contraindications	① Other neurological diseases that affect cognition (excluding AD/PD/FTD); ② Severe internal/common carotid artery stenosis ≥ 70%; ③ Head–neck malignancy or prior deep cervical lymph node dissection; ④ High bleeding risk: platelets < 100 × 10⁹/L; heparin within the past 48 hours with APTT ≥ 35 seconds; warfarin with INR > 1.7; ⑤ Uncontrolled infections (e.g., HIV, syphilis).	MRI‐based exclusions such as >4 cerebral microbleeds, cortical superficial siderosis, or marked cerebrovascular pathology; stroke/TIA/seizure within the past 12 months; patients on anticoagulation are typically excluded (high intracranial hemorrhage risk).	Similar to lecanemab: > 4 microbleeds, cortical superficial siderosis, significant vascular cognitive impairment, etc.	Drug hypersensitivity; relative contraindications such as severe cardiac conduction block or active peptic ulcer—follow the prescribing information and guidelines.	Drug hypersensitivity; alkalinized urine states (which can raise serum drug levels), etc.—follow the prescribing information.
Special populations and risk stratification	① Use caution in anatomically high‐risk cases (prior neck surgery/radiation, vascular variants, neural variants); ② Not suitable for patients with high bleeding risk/on anticoagulation or with severe carotid disease; ③ Bilateral surgery is routine, unilateral may be considered in select cases.	*APOE* genotyping recommended; *APOE* ε4 homozygotes have the highest ARIA risk; bleeding risk is heightened when combined with anticoagulants/thrombolytics and warrants special caution.	*APOE* genotyping recommended; perform ARIA risk stratification and management per Appropriate Use Recommendations (AUR).	*APOE* stratification not required; focus on managing adverse drug reactions and drug–drug interactions.	*APOE* stratification not required; monitor renal function (dose adjustment as needed) and drug–drug interactions.
Implementation setting / resource requirements	Microsurgery + intraoperative lymphatic mapping (ICG, etc.) + perioperative evaluation; requires surgeons experienced in LVA and specialists capable of MRI‐based functional assessment	Intravenous access, infusion center, radiology (serial MRI), with coordinated ARIA management by emergency and neurology teams	Same as left (monthly infusions + MRI monitoring), plus a process for “stop‐treatment evaluation” once plaque clearance is achieved	Outpatient prescribing and follow‐up (oral/patch)	Outpatient prescribing and follow‐up (oral)
Discontinuation / termination rules	Postoperative recommendations: beginning on day 3, if no bleeding is present, administer low‐dose rivaroxaban anticoagulation for 3–6 months with close surveillance for oozing/swelling; if cervical hematoma/active bleeding or definite neural injury occurs, temporarily suspend/adjust anticoagulation and sedatives/analgesics per standard surgical‐complication protocols and prioritize management of the complication.	Hold or discontinue for severe/symptomatic ARIA per AUR; no fixed treatment end point is specified in routine practice.	Consider discontinuation once amyloid PET demonstrates plaque clearance (commonly at 12–18 months).	Do not discontinue mechanically for “disease worsening” alone (NICE advises against stopping solely on the basis of severity).	Individualized assessment; adjust or discontinue if renal function changes or adverse reactions occur.
Concomitant use with other treatments	Explicitly continue preoperative medications (cholinesterase inhibitors/NMDA receptor antagonists, etc.) together with rehabilitation, sleep management, and lifestyle measures. If considering combination with anti‐Aβ mAbs (lecanemab/donanemab), do so within a clinical study and evaluate the potential conflict between ARIA risk and anticoagulation.	May be combined with AChEIs/memantine; not to be combined with aducanumab.	May be combined with AChEIs/memantine; follow AUR and prescribing information.	Commonly combined with memantine.	Commonly combined with AChEIs (for moderate–severe AD).
Regulatory status / evidence and accessibility	The surgery is innovative/exploratory; implemented at multiple domestic centers with short‐ to mid‐term improvement signals; large‐sample randomized controlled evidence and long‐term safety data are still lacking. Multiple clinical trials (ChiCTR and ClinicalTrials.gov) were registered/launched in 2024‐2025; see Table 5.	Approved in the United States for “early AD,” with AUR and an MRI monitoring pathway.	Approved in the United States in 2024 for “early AD,” with AUR and allowance for “discontinuation after plaque clearance.”	Long‐marketed symptomatic therapy (routinely recommended in guidelines across many countries).	Long‐marketed symptomatic therapy (commonly used for moderate–severe AD).
Ref.	^45^, ^47^	^31^	^30^	^6^, ^74^	^5^, ^75^

Abbreviations: Aβ, amyloid beta; AChEI, acetylcholinesterase inhibitor; AD, Alzheimer's disease; ADAS‐Cog, Alzheimer's Disease Assessment Scale Cognitive subscale; *APOE*, apolipoprotein E; APTT, activated partial thromboplastin time; ARIA, amyloid‐related imaging abnormality; CDR, Clinical Dementia Rating; CSF, cerebrospinal fluid; CT, computed tomography; CTA, computed tomography angiography; dcLVA, deep cervical lymphaticovenous anastomosis; DWI, diffusion weighted imaging; FAPI, fibroblast activation protein inhibitor; FDG, fluorodeoxyglucose; FLAIR, fluid‐attenuated inversion recovery; FTD, frontotemporal dementia; GFAP, glial fibrillary acidic protein; GRE, gradient echo; HIV, human immunodeficiency virus; ICG, indocyanine green; INR, international normalized ratio; LVA, lymphaticovenous anastomosis; mAb, monoclonal antibody; MCI, mild cognitive impairment; MMSE, Mini‐Mental State Examination; MoCA, Montreal Cognitive Assessment; MRI, magnetic resonance imaging; NfL, neurofilament light chain; NIA‐AA, National Institute on Aging–Alzheimer's Association; NICE, National Institute for Health and Care Excellence; NMDA, N‐methyl‐D‐aspartic acid; PD, Parkinson's disease; PET, positron emission tomography; p‐tau, phosphorylated tau; SWI, susceptibility‐weighted imaging; TIA, transient ischemic attack.

Patients meeting the above core criteria should undergo rigorous risk evaluation. Based on the literature, the main contraindications for dcLVA include:

1. Definitive non‐AD or mixed dementia causes: For example, dementia with Lewy bodies (DLB) or vascular dementia (VaD). The main pathogenic mechanisms of DLB do not overlap with dcLVA's target (extracellular Aβ/tau clearance), so theoretical benefit is unclear.^36^ In VaD, pathology is driven by cerebrovascular disease (small vessel sclerosis, lacunar infarcts, white matter ischemia/demyelination, microbleeds, etc.) and chronic hypoperfusion causing network disruption; the core issues are perfusion–metabolism imbalance and structural damage, not extracellular protein clearance. Enhancing lymphatic outflow cannot repair vascular lesions or hypoperfusion.^37^
2. Severe carotid artery stenosis (≥ 70%): Severe stenosis of the internal or common carotid artery suggests hemodynamic instability in the carotid sheath and an increased risk of cerebral ischemia. Under microscopic cervical operation, severe carotid stenosis raises the risk of perioperative complications and embolism.^38^
3. History of head/neck malignancy or neck dissection: Previous head/neck tumor surgery or radiation can cause lymphatic anatomical disruption and extensive fibrosis. This makes microsurgical anastomosis difficult and increases the risk of thrombosis/failure at the anastomosis and of chyle leak. Head/neck cancer survivors often have head/neck lymphedema, indicating that local lymphatic return has already been significantly altered, making “enhanced brain–deep cervical drainage” difficult to achieve.^39^
4. High bleeding risk: Platelet count < 100 × 10^9^/L; heparin use within 48 hours with activated partial thromboplastin time (APTT) ≥ 35 seconds; warfarin international normalized ratio (INR) > 1.7. dcLVA is a supermicrosurgical procedure with high bleeding risk: the anastomosis diameter is only 0.3 to 0.8 mm, and any continuous oozing can obscure the transected ends and increase thrombosis and failure rates. Neurosurgical and microsurgical guidelines typically require a platelet count ≥ 100 × 10^9^/L (higher than the ≥ 50 × 10^9^/L used in many other surgeries), and anticoagulation must be managed (for example, stopping intravenous heparin 4–6 hours preoperatively with rechecking APTT/anti‐Xa, and ensuring warfarin INR ≤ 1.5–1.7).^40^, ^41^
5. Uncontrolled infection (e.g., human immunodeficiency virus [HIV], syphilis): Active infection significantly increases the risk of surgical site infection and poor wound healing. Uncontrolled HIV (high viral load, immunosuppression) further raises surgical site infection and perioperative complication rates. Such infections should be controlled and immune status optimized before elective surgery.^42^
6. Advanced AD (CDR global score = 3 or Functional Assessment Staging Tool ≥ 7 with significant dependence): Patients with end‐stage AD typically have very limited remaining functional reserve. Even if improved drainage clears metabolic waste, it is unlikely to reverse existing brain damage, so benefit may be minimal. Moreover, these patients often have high anesthesia and surgical risk and low potential for quantifiable benefit.^43^ They are not recommended for dcLVA, especially if they also have severe brain atrophy or systemic comorbidities (which can indicate lymphatic sclerosis and reduced flow that impair anastomotic success).^44^

It is noteworthy that dcLVA is a one‐time, mechanism‐oriented “pathway enhancement” surgery (see Table 3). It aims to increase the overall clearance throughput of the brain–meningeal–deep cervical chain for Aβ, tau, and other metabolites. Its follow‐up burden is lower than that of monoclonal antibody therapies, which require long‐term infusions and serial MRI monitoring. However, current evidence comes only from small‐sample, short‐term single‐arm studies (the lowest level of evidence), and randomized controlled trials are needed for validation. In terms of pharmacotherapy, lecanemab and donanemab have shown moderate effect sizes and the strongest evidence in early AD, whereas cholinesterase inhibitors and memantine provide symptomatic benefits and are widely accessible but do not alter disease progression. Thus, for early AD, priority should be given to anti‐Aβ antibodies and symptomatic drugs. For moderate‐to‐severe AD or patients who have failed ≥ 6 months of standard therapy and have evidence of impaired brain–deep cervical drainage, dcLVA may be considered (after thorough risk assessment) as a feasibility/safety intervention. Both approaches carry distinct risks: antibodies are mainly associated with amyloid‐related imaging abnormalities (ARIAs), while dcLVA carries perioperative risks. Future studies may explore sequential or combined strategies, but no definitive evidence exists yet.

**TABLE 3:** Comparison of dcLVA to current treatments for Alzheimer's disease.

Approach	Mechanism of action	Evidence / main efficacy	Administration / implementation and monitoring	Advantages	Limitations / risks
dcLVA	Establishes, via supermicrosurgery, a bypass between deep cervical lymphatic vessels/lymph nodes and cervical veins to enhance clearance of metabolic wastes (Aβ, p‐tau, etc.) along the brain–meninges–deep cervical lymphatic pathway.^8^, ^10^, ^55^, ^56^, ^76^	A prospective single‐arm cohort (*n* = 26) reported a median MMSE increase of ≈ 3 points at 1 month post‐op (IQR 0–6) with ≈ 60% of caregivers noting overall improvement; CSF biomarkers trended downward without reaching significance; postoperative complications were rare and reversible.^51^ Single‐case reports showed cognitive‐scale improvement at 5 weeks.^52^ Multiple trials are registered/ongoing (NCT06530732, NCT06448975; several ChiCTR studies).^47^	Supermicrosurgical anastomosis under general anesthesia; pre‐/post‐operative monitoring recommended with MRI, cognitive scales, and plasma and/or CSF biomarkers.^45^, ^51^	One‐time procedure; can be combined with medications; theoretically augments multitarget clearance (Aβ/tau); does not rely on long‐term infusions or frequent infusion visits.^8^, ^10^, ^51^, ^76^	Low evidence quality (no RCTs, short follow‐up, small samples); requires general anesthesia and microsurgery with perioperative risks (delirium/bleeding/infection); long‐term efficacy and mechanisms remain to be validated.^47^, ^51^, ^52^, ^56^
Lecanemab (leqembi, anti‐Aβ protofibril antibody)	Clears cerebral Aβ protofibrils/oligomers, reducing amyloid burden.^77^	Phase III CLARITY‐AD (*n* = 1795): 18‐month CDR‐SB difference −0.45 (1.21 vs. 1.66); secondary endpoints (ADAS‐Cog14/ADCOMS/ADCS‐MCI‐ADL) also improved; ARIA‐E ≈ 12.6%, infusion‐related reactions 26.4%^77^; AUR recommends *APOE* ε4 genotyping and strict MRI monitoring.^33^	10 mg/kg IV every 2 weeks; MRI monitoring for ARIA at baseline and early/during treatment; use caution if combined with anticoagulants/thrombolytics.^33^, ^77^	Statistically significant slowing across multiple clinical scales; well‐defined mechanism with concordant biomarker evidence.^77^	Moderate absolute effect size; requires long‐term, repeated infusions and imaging surveillance; elevated ARIA risk (especially in *APOE* ε4 homozygotes).^33^, ^77^
Donanemab (Kisunla, anti‐Aβ plaque antibody)	Rapid clearance of amyloid plaques with a “time‐limited” strategy allowing discontinuation once a predefined clearance threshold is met.^78^, ^79^	Phase III TRAILBLAZER‐ALZ 2: primary endpoint iADRS difference 3.25 in the low/medium‐tau subgroup (≈ 35% slowing); CDR‐SB difference ≈ −0.67 (low/medium tau); a subset achieved plaque‐clearance criteria within 12 months [14]. FDA approved; 2025 label updates a stepwise dose‐escalation to lower ARIA‐E incidence; prescribing information reports overall ARIA ≈ 29%, ARIA‐E ≈16%.^78^	IV infusion every 4 weeks; 2025 label recommends stepwise titration 350→700→1050→1400 mg, then maintenance; MRI monitoring for ARIA is required; use caution with anticoagulants/thrombolytics.^78^	Improvements across clinical scales and imaging/blood biomarkers; time‐limited therapy possible for some patients; monthly dosing.^78^, ^79^	ARIA risk and monitoring burden; weaker effect in high‐tau populations; cost and accessibility concerns.^78^, ^79^
Cholinesterase inhibitors (donepezil/galantamine/rivastigmine)	Inhibit acetylcholinesterase, enhance cholinergic neurotransmission, and provide symptomatic improvement.^7^, ^80^	Systematic reviews and randomized controlled trials show small‐to‐moderate improvements in cognition, activities of daily living, and global impression in mild‐to‐moderate AD.^7^, ^80^	Oral or transdermal patch (rivastigmine); initiate at a low dose and titrate upward; monitor for gastrointestinal adverse effects, weight loss, and bradycardia.^7^, ^80^	Oral/patch formulations; widely accessible; relatively low cost; extensive clinical experience.^7^, ^80^	Symptomatic only; limited magnitude of benefit; adverse effects include nausea, vomiting, and bradycardia.^7^, ^80^
Memantine (NMDA receptor antagonist)	Reduces glutamate‐mediated excitotoxicity, providing symptomatic improvement.^5^	Moderate‐to‐severe AD RCT: over 28 weeks, slowed deterioration in function/behavior; overall well tolerated.^5^	Oral; titrate up to 20 mg/day; monitor for dizziness, constipation, etc.^5^	Oral administration; generally well tolerated; additive symptomatic benefit with cholinesterase inhibitors.^5^	Symptomatic only; limited efficacy in early disease.^5^

Abbreviations: Aβ, amyloid beta; AD, Alzheimer's disease; ADAS‐Cog, Alzheimer's Disease Assessment Scale Cognitive subscale; ADCOMS, Alzheimer's Disease Composite Score; *APOE*, apolipoprotein E; ARIA, amyloid‐related imaging abnormality; AUR, Appropriate Use Recommendations; CDR‐SB, Clinical Dementia Rating Sum of Boxes; CSF, cerebrospinal fluid; dcLVA, deep cervical lymphaticovenous anastomosis; FDA, US Food and Drug Administration; iADRS, integrated Alzheimer's Disease Rating Scale; IQR, interquartile range; MCI, mild cognitive impairment; MMSE, Mini‐Mental State Examination; MRI, magnetic resonance imaging; NMDA, N‐methyl‐D‐aspartic acid; p‐tau, phosphorylated tau; RCT, randomized controlled trial.

### 3.2 Standardized surgical technique and research protocols for dcLVA

#### 3.2.1 Standardized procedure and ethical framework for dcLVA in AD

**FIGURE 2:**
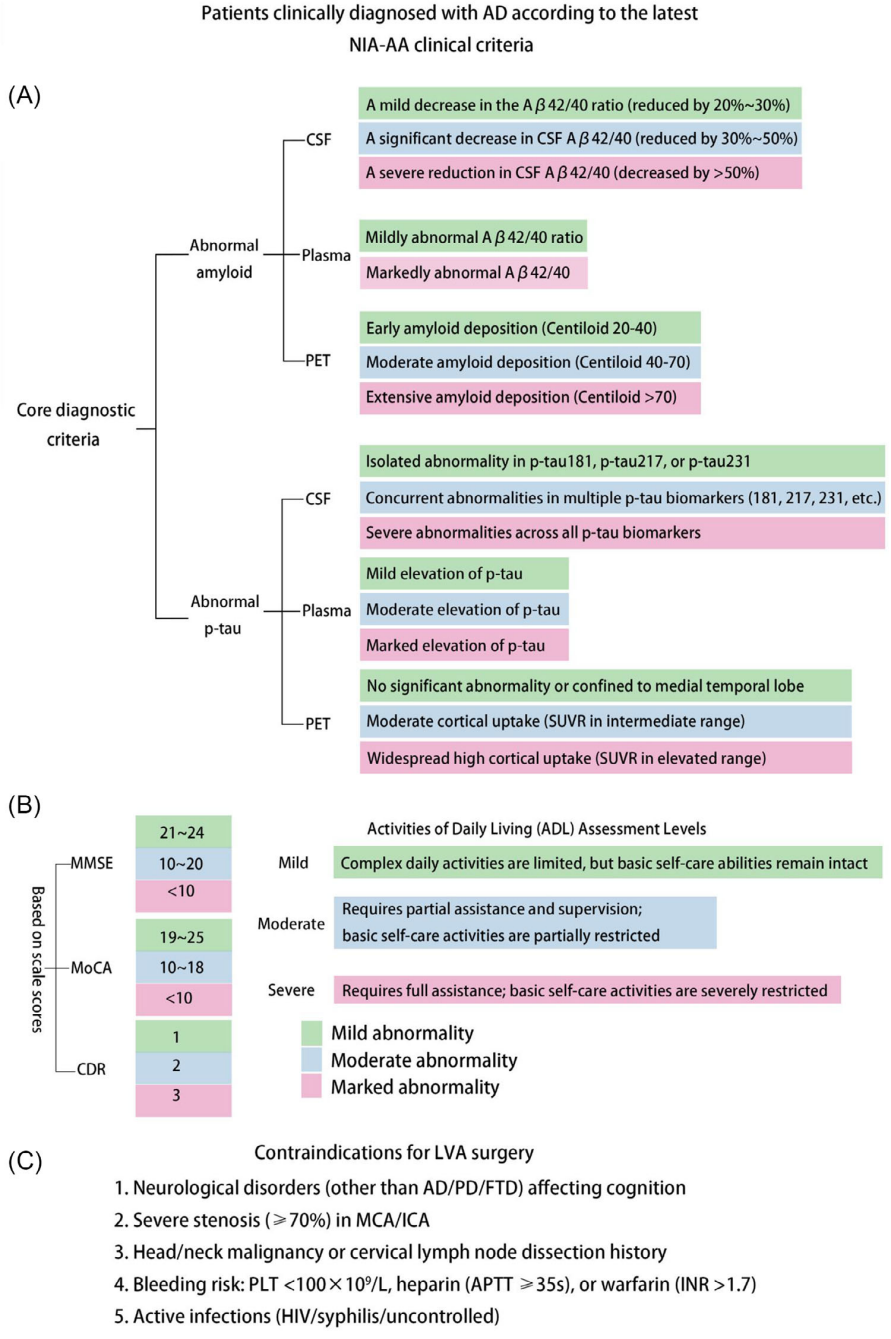
Diagnostic criteria for AD patients, scale scores, ADL assessment, and contraindications for LVA surgery.^45^, ^46^ A, Light green indicates mildly abnormal indicators, light blue moderately abnormal, and pink markedly abnormal. Diagnosis of mild AD requires only one core biomarker to be mildly abnormal; diagnosis of moderate AD requires both core biomarkers to be abnormal with at least one moderately abnormal; diagnosis of severe AD requires both core biomarkers to be markedly abnormal. B Light green denotes mild AD, light blue moderate AD, and pink severe AD. C, Contraindications for LVA surgery. Aβ, amyloid beta; AD, Alzheimer's disease; ADL, activities of daily living; APTT, activated partial thromboplastin time; CDR, Clinical Dementia Rating; CSF, cerebrospinal fluid; FTD, frontotemporal dementia; HIV, human immunodeficiency virus; ICA, internal carotid artery; INR, international normalized ratio; LVA, lymphaticovenous anastomosis; MCA, middle cerebral artery; MMSE, Mini‐Mental State Examination; MoCA, Montreal Cognitive Assessment; NIA–AA, National Institute on Aging–Alzheimer's Association; PD, Parkinson's disease; PET, positron emission tomography; PLT, platelet count; p‐tau, phosphorylated tau; SUVR, standardized uptake value ratio.

According to the Chinese Expert Consensus on Lymphatic Surgery for Alzheimer's Disease (2025 Edition),^45^ dcLVA requires a prespecified workflow encompassing preoperative assessment, eligibility confirmation, intraoperative microanastomosis, and postoperative surveillance. As outlined in Figure 2, preoperative assessment includes confirmation of AD pathology by ≥ 2 abnormal core biomarkers—for example, low CSF/plasma Aβ42 or reduced Aβ42/40 ratio, elevated phosphorylated tau (p‐tau), and/or positive amyloid positron emission tomography (PET)—together with standardized cognitive staging using MMSE, Montreal Cognitive Assessment (MoCA), CDR, and Alzheimer's Disease Assessment Scale Cognitive subscale. Candidates are typically moderate‐to‐severe (MMSE ≤ 20 or CDR ≥2) and should additionally demonstrate objective evidence of impaired brain–meningeal–deep cervical lymphatic drainage on imaging, aligning with the mechanistic rationale for dcLVA.^46^ Given that dcLVA is still in an early exploratory stage, it is recommended to perform it only within a regulated research framework. All procedures must have institutional ethics committee approval and comply with the Declaration of Helsinki. Written informed consent should explicitly state that dcLVA is exploratory, with potential risks (such as anastomotic failure, bleeding, infection, etc.), alternative treatments, expected benefits, and perioperative/postoperative monitoring plans outlined. With the current limited evidence, dcLVA should not be used as a standalone treatment; it should be integrated into multidisciplinary management alongside guideline‐driven medical therapy (including anti‐Aβ antibodies when indicated), cognitive rehabilitation, and lifestyle interventions. Objective efficacy endpoints and safety monitoring protocols should be prespecified.^47^ When feasible, dcLVA should be implemented in prospective registries or clinical trials to ensure data quality and comparability.

#### 3.2.2 Overview of dcLVA technique and reporting recommendations

**FIGURE 3:**
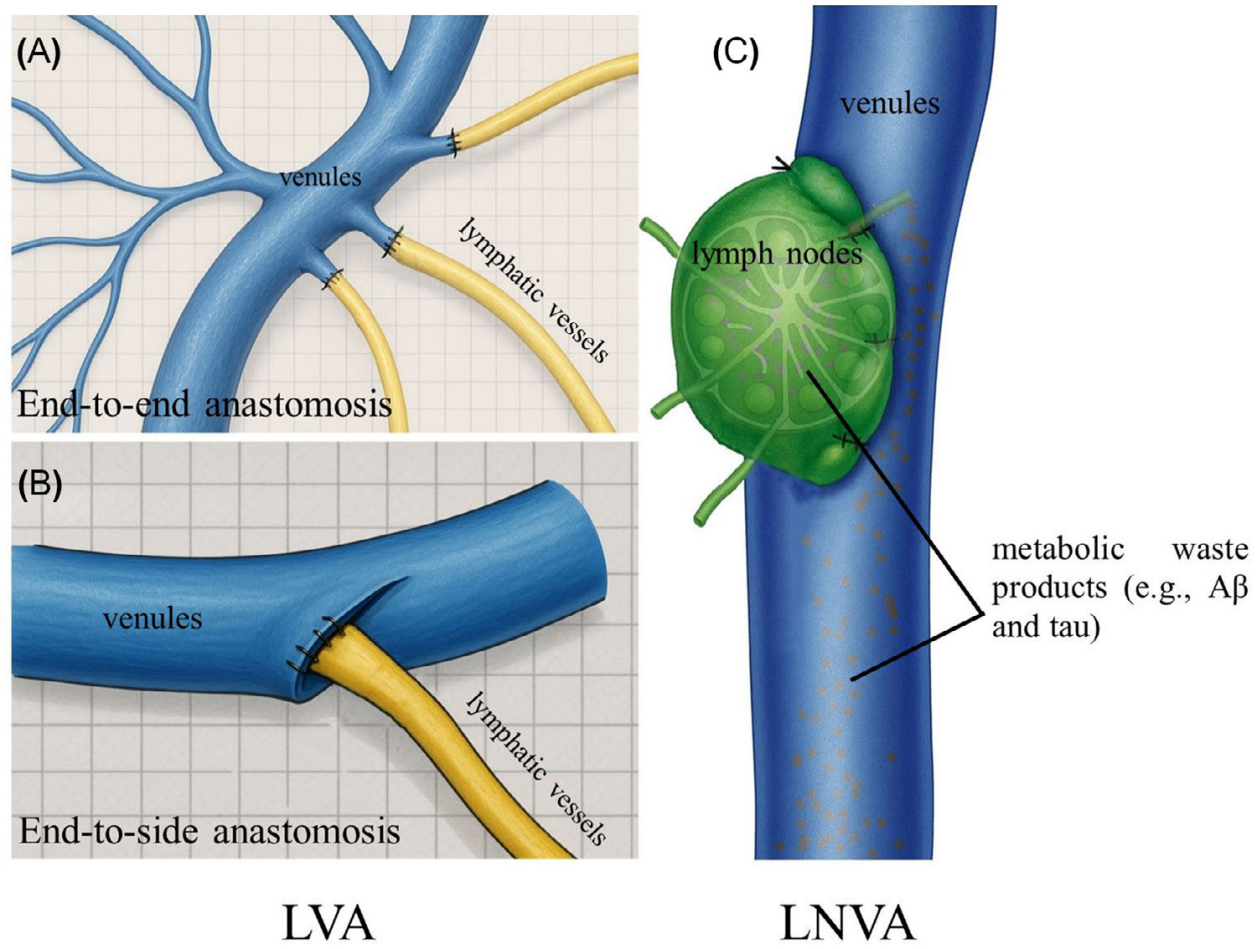
Schematic diagram of LVA and LNVA surgery. A, Directly anastomosing the severed ends of two vessels to form a continuous channel. B, Anastomosing the severed end of the lymphatic vessel to the side wall incision of the vein, maintaining the continuity of the channel. C, Anastomosing the capsule of the lymph node to the adjacent vein to establish a direct drainage channel for lymphatic fluid into the venous system. LNVA, lymph node–venous anastomosis; LVA, lymphaticovenous anastomosis.

**FIGURE 4:**
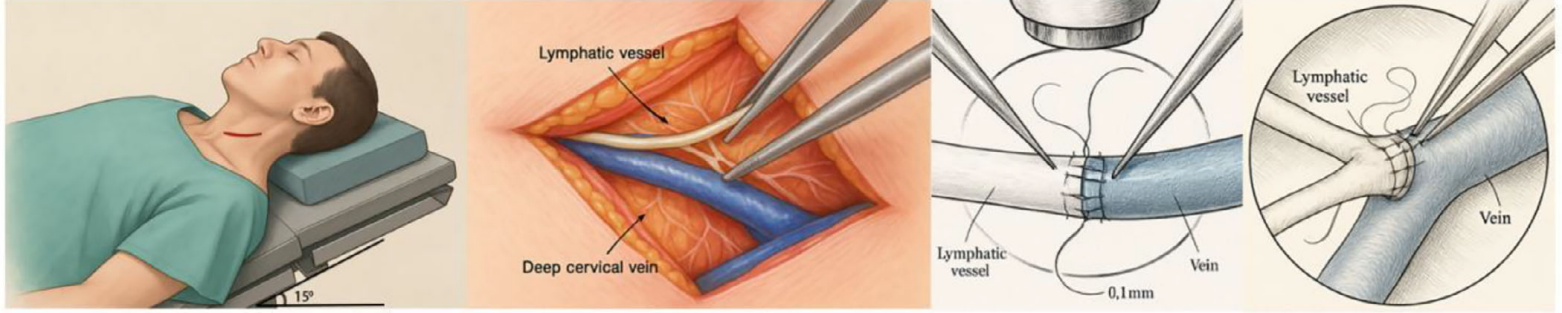
Schematic diagram of deep cervical lymphaticovenous anastomosis surgery.

For the purpose of this review, the operative description is limited to a conceptual overview. In LVA surgery, a low‐resistance lymphatic–venous bypass is created by microsuturing collecting lymphatic channels (≈ 0.1–0.4 mm in diameter) to adjacent small venules. The anastomoses are typically configured as end‐to‐end or end‐to‐side (Figures 3 and 4), chosen according to vessel size match and flow directionality.^25^, ^48^ Lymph node–venous anastomosis (LNVA) is a variant that connects a lymph node unit to a vein and is considered a distinct procedure within lymphatic–venous surgeries.^49^ Both dcLVA and LNVA are performed under general anesthesia with the patient in the supine position and the neck mildly extended. A transverse incision of ≈ 2 to 3 cm is made along the posterior border of the sternocleidomastoid muscle (or at the thyroid cartilage level). The subcutaneous tissue and platysma muscle are incised in sequence to expose the sternocleidomastoid fascia. The surgeon carefully dissects along this plane to identify the lymphatic structures and corresponding small veins. Intraoperatively, indocyanine green (ICG) fluorescence imaging is often used to help visualize lymphatic vessels and nodes.^50^ Anastomoses are performed under the microscope with fine sutures (e.g., 11‐0 or 12‐0 nylon). Patency is assessed using ICG fluorescence; if dye rapidly travels from the lymphatic channel into the venous system, the anastomosis is deemed successful. After achieving hemostasis, the incision is closed in layers, and placement of a surgical drain is at the surgeon's discretion.

To improve study comparability and reporting standardization, we recommend that future clinical reports should include: (1) anastomosis type and number, recipient vein category and approximate caliber; (2) methods for localization and patency verification (e.g., ICG transit); (3) technical success rate (intraoperative flow across anastomoses) and early failures; and (4) a structured profile of complications (hematoma, infection, chyle leak, transient neural irritation, etc.), with prespecified imaging and clinical follow‐up endpoints(see Tables 2 and 3). Routine perioperative management (anesthesia, skin preparation, suturing, drains, etc.) should follow standard protocols and is not detailed here.

### 3.3 Clinical evidence and patient outcomes after dcLVA

Published evidence on dcLVA for AD remains very limited. To date, it consists of one single‐center prospective single‐arm exploratory cohort study (*n* = 26) and a small number of case reports. To avoid narrative bias, we summarize only the available objective data, corresponding to Table 4 (which details study design, sample size, follow‐up timepoints, and main outcomes) and Table 5 (registered/in‐progress trials).

**TABLE 4:** Clinical evidence and outcomes of dcLVA in treating Alzheimer's disease.

Patient type / inclusion criteria	Sample size	Follow‐up duration	Main findings	Conclusion	Response rate	Ref.
AD confirmed by NIA–AA biomarker and clinical diagnostic criteria; predominantly moderate‐to‐severe	*n* = 26	1 month post‐op	Approximately 60% of caregivers reported overall symptomatic improvement; median MMSE increased from 3 to 5 (*p* = 0.022); MoCA ↑15% and NPI ↓42% did not reach statistical significance; CSF Aβ and tau showed downward trends without significance; two cases of shoulder‐abduction difficulty, both resolved on follow‐up	Short‐term statistically significant MMSE improvement with acceptable safety; larger, longer‐term studies are needed for validation	≈ 60% (caregiver‐reported overall improvement rate)	^51^
Severe AD case; preoperative assessment included PET and clinical scales	*n* = 1	5 weeks	Improvement on clinical scales; decreased overall tau PET burden (more pronounced in the left temporal lobe); increased metabolism on ^18^F‐FDG PET	Very early imaging and clinical improvements suggest potential efficacy	—	^52^
58‐year‐old woman with severe AD and BPSD; Aβ PET positive	*n* = 1	4 months	Marked improvement in MMSE/MoCA; ^18^F‐AV‐45 PET/CT showed decreased cerebral amyloid burden	Suggests possible improvement in cognition and imaging biomarkers	—	^53^
74‐year‐old woman with AD/mixed dementia; refractory to medication	*n* = 1	3 months	MMSE improved from 0 to 5; improvements in language and self‐care; no care‐related complications observed	Suggests short‐term postoperative functional improvement	—	^27^

Abbreviations: Aβ, amyloid beta; AD, Alzheimer's disease; BPSD, behavioral and psychological symptoms of dementia; CSF, cerebrospinal fluid; CT, computed tomography; dcLVA, deep cervical lymphaticovenous anastomosis; FDG, fluorodeoxyglucose; MCI, mild cognitive impairment; MMSE, Mini‐Mental State Examination; MoCA, Montreal Cognitive Assessment; NIA‐AA, National Institute on Aging–Alzheimer's Association; NPI, Neuropsychiatric Inventory; PET, positron emission tomography.

**TABLE 5:** Registered and ongoing clinical trials of lymphatic–venous anastomosis for Alzheimer's disease.

Registration No.	Title	Lead institution / region	Design / phase / status	Target sample size / enrollment	Primary endpoint(s)
ChiCTR2400084617	Randomized controlled clinical trial of deep cervical lymphatic–venous anastomosis combined with lymphatic pedicle excision for AD	Shanghai Ninth People's Hospital, Shanghai Jiao Tong University School of Medicine; China	Randomized controlled; phase not specified; registered on 2024‐05‐21	Not disclosed	Not disclosed
ChiCTR2400089883	Single‐center, prospective, single‐arm exploratory study: deep cervical lymphatic–venous anastomosis to improve neurological function in AD	Department of Neurosurgery, First Affiliated Hospital of Army Medical University (Southwest Hospital); China	Single‐arm prospective; registered on 2024‐09‐19	Prospective cohort overlapping with^51^; *n* ≈ 26 (per published report)	Neuropsychological scales, CSF biomarkers, etc.
ChiCTR2400092975	Evaluation of comprehensive diagnosis and treatment outcomes for AD based on bilateral deep cervical lymphatic–venous anastomosis	Zhengzhou Central Hospital; China	Prospective cohort; registered on 2024‐11‐26	Not disclosed	Composite outcomes including CDR‐SB (per hospital page and reports)
ChiCTR2400093030	Clinical efficacy of deep cervical lymphatic–venous anastomosis in patients with type 2 diabetes mellitus and AD	Zhengzhou Central Hospital; China	Prospective; registered on 2024‐11‐27	Not disclosed	Metabolic and cognitive endpoints (per registry entry)
ChiCTR2400094603	Randomized controlled clinical trial of deep cervical lymphatic–venous anastomosis for Alzheimer's disease	Zunyi First People's Hospital; China	Randomized controlled; registered on 2024‐12‐25	Not disclosed	CDR‐SB, etc.
NCT06448975	Exploratory study of deep cervical lymphatic–venous bypass (LVB) for treating AD	Affiliated Hospital of Jiangnan University; China	Prospective, two‐arm; target enrollment 30; start 2024‐05‐31; estimated completion 2026‐07‐01; recruiting (per third‐party aggregator)	30	Cognitive scales; imaging/biomarker measures (per registry entry)
NCT06530732(DIVA)	Pilot study of deep cervical lymphatic–venous anastomosis for AD (including standard‐of‐care drug comparator, e.g., lecanemab)	Zhejiang Provincial People's Hospital; China	Randomized controlled; Phase 3 (per third‐party registry page); primary endpoint CDR‐SB at 12 months; recruiting	Not disclosed	CDR‐SB at 12 months; protocol includes MRI and lumbar puncture with gadolinium contrast, etc.
NCT07058129(CLEAN‐AD)	Multicenter real‐world registry study of deep cervical lymphatic–venous anastomosis for AD	Multicenter (China)	Prospective registry cohort; planned 814 participants across 40 centers; follow‐up to 24 months; status per registry	814 (planned)	Primary: 12‐month change in CDR‐SB; Secondary: NPI, ADCS‐ADL, MMSE, ADAS‐Cog13, Aβ PET CT, etc.
NCT07073066(CLEAN‐AD RCT)	Deep cervical lymphatic–venous anastomosis plus standard of care vs. standard of care: randomized, open‐label, blinded‐endpoint trial in moderate‐to‐severe AD	Capital Medical University et al.; China	Multicenter, randomized, open‐label, blinded‐endpoint; recruitment to commence / registry update forthcoming	Not disclosed	12‐month change in CDR‐SB; safety

Abbreviations: Aβ, amyloid beta; AD, Alzheimer's disease; ADAS‐Cog, Alzheimer's Disease Assessment Scale Cognitive subscale; ADCS‐ADL, Alzheimer's Disease Cooperative Study Activities of Daily Living; CDR‐SB, Clinical Dementia Rating Sum of Boxes; CSF, cerebrospinal fluid; CT, computed tomography; MMSE, Mini‐Mental State Examination; MRI, magnetic resonance imaging; NPI, Neuropsychiatric Inventory; PET, positron emission tomography; RCT, randomized controlled trial.

In the single‐arm cohort study, all patients met the National Institute on Aging–Alzheimer's Association clinical and biomarker criteria for AD. The procedure was a modified deep cervical LVA (changing from a “lymphatic vessel–vein” to a “lymphatic flap–vein” anastomosis). One month postoperatively, ≈ 60% of caregivers reported some degree of overall symptom improvement. The MMSE score showed a statistically significant increase from baseline—the study reported a median increase of ≈ 3 points (interquartile range 0–6, *p* = 0.022). MoCA and the Neuropsychiatric Inventory (NPI) did not show significant changes at the group level; however, 15% of patients had MoCA increases and 42% had reductions in NPI scores. CSF biomarkers (Aβ42, Aβ40, p‐tau, total tau, etc.) showed overall downward trends, though none reached statistical significance. In terms of safety, no severe surgery‐related adverse events were observed; only two patients experienced postoperative difficulty with shoulder abduction, which gradually resolved on follow‐up.^51^ These findings, assessed at 1 month, suggest early signals of effect, but longer follow‐up is needed to determine durability and clinical significance.

Case reports provide additional detail on objective improvements. The first reported case of a “cervical shunting procedure to unclog the cerebral lymphatic system” (published in *General Psychiatry*) showed concordant improvements in scales and imaging at 5 weeks post‐op: MMSE rose from 5 to 7, CDR‐SB dropped from 10 to 8, and Global Deterioration Scale (GDS) fell from 9 to 0. Concurrently, ^18^F‐FDG (fluorodeoxyglucose) PET showed globally increased brain glucose metabolism (especially in the right frontal lobe), and tau PET showed decreased whole‐brain signal. That report also provided the patient's biomarker‐confirmed diagnosis and ethics registration details.^52^ Another open‐access case report performed quantitative ^18^F‐AV‐45 (florbetapir) amyloid PET at 4 months post‐op: standardized uptake value ratios (SUVRs) in regions such as the frontal, parietal, temporal lobes, and posterior cingulate decreased (for example, frontal SUVR from 1.53 to 1.27). Corresponding Centiloid (CL) values declined across multiple brain regions (e.g., frontal CL from ≈ 102.8 to 55.2), consistent with reported improvements in communication and daily function. That report also detailed the context of the patient's severe AD and poor drug response, as well as perioperative management.^53^ A further case in the *Journal of Alzheimer's Disease Reports* described an MMSE increase from 0 to 5 at 3 months postop, with reported improvements in language and self‐care, and no nursing‐related complications, suggesting preliminary feasibility under strict perioperative management.^27^

It must be emphasized that these early results should be interpreted with caution. First, follow‐up was mostly limited to 1 month, with very few cases beyond 3 to 4 months, making it difficult to infer mid‐ and long‐term efficacy and safety. Second, studies to date have been primarily uncontrolled, single‐arm cohorts or case reports, which are susceptible to regression to the mean, placebo effects, and selection bias. Third, there is heterogeneity in naming and technique (e.g., dcLVA, lymphatic‐venous bypass, cervical shunting, deep cervical venous lymphatic anastomosis, etc.) and in patient baseline severity, all of which limit cross‐study comparisons and meta‐analysis. Therefore, we present Table 4 with results as originally reported, without pooling effect sizes. Future research urgently needs preregistered trials with unified primary endpoints; standardized reporting of effect sizes (with 95% confidence intervals), MCID responder rates, and structured safety events; and systematic inclusion of imaging (Aβ PET, tau ‐PET, and FDG PET) and fluid (CSF/plasma Aβ and p‐tau) biomarkers, validated alongside standardized clinical outcomes (such as CDR‐SB, ADCS‐ADL, and NPI). Recent review articles have also emphasized that current clinical evidence consists mostly of scattered cases, with no rigorously validated randomized trials, and caution against overinterpreting “rapidly spreading” social media narratives.^47^

Regarding ongoing studies, ClinicalTrials.gov lists at least two prospective studies of dcLVA/lymphatic–venous bypass for AD: the DIVA pilot study led by Zhejiang Provincial People's Hospital (NCT06530732) and an exploratory study led by the Affiliated Hospital of Jiangnan University (NCT06448975). Both plan to assess longer term clinical scales and imaging and/or fluid biomarkers, aligning with our suggested endpoints (e.g., 12‐month CDR‐SB). The Chinese Clinical Trial Registry also lists several related studies, including randomized controlled trials and combined treatment pathway evaluations. We will continue to follow these trials and their outcomes to improve the evidence base and generalizability.

## 4 PROSPECTS AND CHALLENGES OF dcLVA IN AD

dcLVA offers a novel therapeutic approach for AD. The procedure is minimally invasive (a 2–3 cm neck incision) with quick recovery; theoretically it avoids direct brain tissue injury from craniotomy, potentially shortening hospital stay and reducing complications.^45^ However, current reports on dcLVA in AD are mostly case narratives and media stories, lacking rigorous clinical evaluation.^47^ Overall, clinical research on dcLVA remains in its infancy, with small‐scale studies (one prospective single‐arm cohort [*n* = 26] and a few case reports) that have very limited samples, no controls, and inconsistent endpoint definitions.^28^, ^51^, ^52^, ^53^ Therefore, although short‐term improvements on standardized scales and imaging are observed, the long‐term efficacy, reproducibility, and true clinical benefit of dcLVA require validation in larger, controlled studies with ≥ 12 month follow‐up; until such data are available, expectations for its effectiveness should remain cautious. Reported adverse events have been few and mostly reversible (such as transient shoulder abduction weakness),^51^ but data on long‐term systemic safety are insufficient and will require systematic monitoring and reporting.

Physiologically, meningeal lymphatic vessels drain CSF and macromolecules from the CNS to the deep cervical lymph nodes in animals and humans, as shown in foundational studies and human MRI/tracer work;^8^, ^9^, ^10^, ^11^, ^15^, ^19^ from there, lymph ultimately enters the venous circulation via the right lymphatic duct/thoracic duct at the venous angles.^54^ dcLVA, therefore, establishes a distal low‐resistance bypass within this existing cervical lymphatic–venous outflow rather than creating a wholly new route.^8^, ^9^, ^10^, ^11^, ^15^ Nevertheless, the quantitative systemic exposure to CNS‐derived proteins (e.g., Aβ, tau) after dcLVA and any long‐term hepatic/renal or hematological consequences remain unknown: to our knowledge, no published study in dcLVA for AD has prospectively and longitudinally monitored comprehensive liver function, kidney function, complete blood count, or inflammatory markers to address this concern.^27^, ^51^, ^52^ Although lymphaticovenous bypass is widely reported as feasible and generally safe in peripheral lymphedema, these series seldom include systematic organ‐function surveillance and are not directly generalizable to AD.^24^, ^49^, ^55^, ^56^ Accordingly, future dcLVA trials and registries should prespecify systemic safety endpoints (baseline and serial comprehensive metabolic panel/liver function tests, renal panels, complete blood count, and C‐reactive protein), track plasma Aβ42/40, p‐tau, neurofilament light chain, glial fibrillary acidic protein, and adjudicate any hepatobiliary/renal adverse events over 12 to 24 months before considering broader clinical adoption.

dcLVA is not suitable for all AD patients. For early, mild AD patients, there is currently no evidence that their benefit would clearly outweigh the risks; for patients in very late‐stage disease with extensive neuronal loss, even if drainage improves waste clearance, it is unlikely to reverse existing brain damage, so therapeutic effect may be limited. Furthermore, most AD patients are elderly and often have comorbidities such as hypertension or diabetes, which increase surgical risk and make frail patients poor surgical candidates.^47^ Patient selection is extremely challenging: one must identify which patients have lymphatic drainage dysfunction leading to inadequate clearance of brain waste to benefit from dcLVA. Currently, methods to assess brain glymphatic system function preoperatively are very limited, although some studies have begun to explore this. For example, DTI‐ALPS has been used to quantify glymphatic activity, and AD patients have been found to have significantly lower DTI‐ALPS indices, suggesting impaired brain glymphatic flow.^57^ Although these imaging methods are still in early exploratory stages and provide new ideas for preoperative screening, further research is needed to validate their clinical utility and how they might optimize patient selection.

Surgery itself also carries potential risks. Although dcLVA is minimally invasive, it is performed under general anesthesia and microscopically, and perioperative cognitive complications are not uncommon in the elderly, especially those with baseline dementia or cognitive impairment.^58^ International consensus now includes perioperative cognitive outcomes (delirium and postoperative neurocognitive disorders up to 12 months) under the category of “perioperative neurocognitive disorders (PND).” Therefore, when discussing the risk–benefit of dcLVA, these outcomes must be considered.^58^ Epidemiological evidence shows that in patients ≥ 65 undergoing non‐cardiac surgery, postoperative delirium can occur at high rates, and a significant proportion of patients experience persistent cognitive decline afterward. Importantly, in AD patients, the occurrence of delirium often accelerates long‐term cognitive decline, suggesting that even a transient acute brain dysfunction episode can have lasting effects on the vulnerable AD brain.^59^, ^60^ Early multicenter studies also indicate a risk of long‐term postoperative cognitive decline in older adults; although the direct causal link between anesthesia type and long‐term cognition is debated, consensus holds that advanced age, pre‐existing cognitive decline, intraoperative physiological fluctuations, and perioperative complications are key risk factors.^61^, ^62^ Based on current evidence, one should not assume that any cognitive risk from anesthesia is necessarily offset by dcLVA's benefits. A more reasonable approach is individualized risk–benefit assessment: dcLVA's potential benefits may only outweigh cognitive risks if the patient has objectively demonstrated brain–glymphatic clearance impairment, has progressive symptoms not controlled by medications and rehabilitation, and has good anesthesia tolerance with controllable systemic risk. Under these conditions, with stringent perioperative prevention and monitoring, the benefits of dcLVA might justify the risks. To minimize risks, guidelines for perioperative delirium prevention should be followed (e.g., American Geriatrics Society guidelines), including preoperative risk stratification (age, baseline cognition, sensory deficits, and polypharmacy), optimizing anesthesia depth and analgesia, minimizing anticholinergic and sedative burden, promoting early mobilization and sleep–wake management, and incorporating standardized neuropsychological assessments and appropriate imaging or biomarker monitoring during follow‐up.^63^

Given the uncertainty about dcLVA's effectiveness, researchers are also exploring other non‐invasive or combined therapies. For example, photobiomodulation (PBM) is an emerging treatment that uses near‐infrared light to modulate microglial phenotypes, thereby reducing neuroinflammation and promoting neuronal survival.^64^, ^65^ Studies have shown that phototherapy targeting neurodegenerative diseases like Parkinson's and AD has shown potential in animal models and preliminary clinical settings, but its clinical efficacy and mechanisms require further study.^66^, ^67^ In addition, dcLVA is not the only therapy; in the future it may be used in combination with medications and rehabilitation exercises.^68^ For example, supplementing dcLVA with anti‐Aβ drugs or cognitive training postoperatively could theoretically provide synergistic treatment across multiple targets, but the safety and efficacy of such combinations need to be evaluated in evidence‐based trials.

In summary, dcLVA introduces a novel treatment concept for AD, but the current evidence base is still very limited. Caution is warranted, and patients and families must be fully informed with thorough risk assessment. Large, multicenter randomized controlled trials and long‐term follow‐up studies are urgently needed to determine the effects of dcLVA on cognitive function, brain pathology, and quality of life. Simultaneously, further research into its mechanisms (for example, its effects on brain lymphatic clearance pathways and neuroinflammation) and potential risks is necessary. Only through rigorous scientific validation can the true value and proper role of dcLVA in AD treatment be established.

## 5 CONCLUSION

Based on the brain–meningeal lymphatic–deep cervical chain clearance axis, dcLVA provides a mechanism‐driven surgical approach for AD. Current evidence, which consists mainly of small prospective single‐arm studies and case reports, suggests that short‐term postoperative cognitive and imaging biomarkers may show improvement signals, and perioperative complications are generally controllable. However, due to lack of control groups, small sample sizes, and short follow‐up durations, long‐term efficacy and systemic safety remain unproven. Furthermore, dcLVA is not appropriate for all patients. Early‐stage patients should first receive evidence‐based pharmacotherapy. For moderate‐to‐severe patients with objective evidence of limited brain–deep cervical drainage who continue to progress despite standard treatment, dcLVA can be considered a research option after thorough informed consent that includes anesthesia‐related risks of PND/delirium. Future work must include multicenter randomized controlled trials with preregistration and standardized primary endpoints (e.g., 12‐month CDR‐SB) along with Aβ/tau PET, CSF/plasma biomarkers, and DTI‐ALPS imaging evaluations. Results should be published transparently using standardized techniques and complication reporting to clarify the true clinical value and proper positioning of dcLVA. Combined or sequential strategies with anti‐Aβ antibodies and rehabilitation should also be tested in trial settings.

## AUTHOR CONTRIBUTIONS

Qingwen Chen: conceptualization; literature search and selection; writing–original draft; writing–review and editing. Qianmei Wen: literature search and selection; formal analysis; writing–original draft. Tao Zhong: writing–original draft. Jian Liu: writing–review and editing. Han Gao: conceptualization; project administration; supervision; writing–review and editing.

## CONFLICT OF INTEREST STATEMENT

The authors declare no conflicts of interest.Author disclosures are available in the supporting information

## ETHICS APPROVAL AND CONSENT TO PARTICIPATE

Not applicable.

## CONSENT FOR PUBLICATION

Not applicable.

## Supporting information



Supporting Information

## Data Availability

Not applicable.
